# Reticulate and Hybrid Speciation is Promoted by Environmental Instability in an Indo‐Pacific Species Complex of Whistlers (Aves: *Pachycephala*)

**DOI:** 10.1111/mec.70018

**Published:** 2025-07-12

**Authors:** Martin Irestedt, Ingo A. Müller, Filip Thörn, Leo Joseph, Johan A. A. Nylander, Benjamin Guinet, Tom van der Valk, Knud Andreas Jønsson

**Affiliations:** ^1^ Department of Bioinformatics and Genetics Swedish Museum of Natural History Stockholm Sweden; ^2^ Department of Zoology Stockholm University Stockholm Sweden; ^3^ Australian National Wildlife Collection National Research Collections Australia, CSIRO Canberra Australia; ^4^ Centre for Palaeogenetics Stockholm Sweden; ^5^ Science for Life Laboratory Stockholm Sweden; ^6^ Natural History Museum of Denmark University of Copenhagen Copenhagen Ø Denmark

**Keywords:** birds, genomics/proteomics, hybridisation, population dynamics, population genetics – empirical, speciation

## Abstract

Genomic studies have revealed introgressive hybridisation as a common phenomenon across the tree of life, particularly among young radiations. As incipient speciation tends to be induced by vicariance events, it is assumed that introgressive hybridisation is more frequent in young radiations in which allopatrically distributed species have a high probability of coming into secondary contact. In this study, we use whole genomic data to investigate spatio‐temporal introgression patterns in a songbird radiation that has colonised a highly dynamic island region in the Indo‐Pacific. Some taxa within this radiation have colonised remote oceanic islands whereas others occur on landmasses and islands in the Sahul region that were periodically connected during Pleistocene periods of lower sea levels. Our results show that introgressive hybridisation has been pervasive within this young radiation, despite prominent plumage differences between taxa. Geographical proximity has been an important factor for hybridisation and we further find that species occupying islands in the environmentally unstable Sahul region exhibit particularly high signatures of introgressive hybridisation. Yet, one species appears to have been shielded from hybridisation, perhaps due to specific ecological specialisations. Finally, we identify a hybrid species on an island where two oceanic radiations meet. Our results also caution against relying solely on analyses that only detect asymmetric introgression when examining systems with complex introgression histories. Collectively, our results support a growing body of literature that suggests that reticulate speciation is more common than previously thought. This has implications for our understanding of species formation and their persistence through time.

## 1 Introduction

Introgressive hybridisation is pervasive across the tree of life (Abbott et al. 2013; Blom et al. 2024; Goulet et al. 2017; Hedrick 2013; Seehausen 2004; Taylor and Larson 2019) but the evolutionary significance of genetic introgression can vary along a continuum from being deleterious to adaptive (Abbott et al. 2013; Adavoudi and Pilot 2022) and in extreme cases even trigger diverse adaptive radiations (Meier et al. 2017; Seehausen 2004). In theory, extensive introgression can almost completely homogenise the autosomal genomes of hybridising species, while specific differences can be retained in small genomic regions (genomic islands) (Feder et al. 2012; Harrison and Larson 2014; Seehausen et al. 2014). The number of empirical examples of this is steadily increasing (e.g., Poelstra et al. 2014; Toews et al. 2016). Introgression may also lead to the formation of entirely new species (Lamichhaney et al. 2018; Ottenburghs 2018; Rosser et al. 2024; van der Valk et al. 2021; Wang et al. 2022) or to speciation reversal when previously isolated lineages are fused (Behm et al. 2010; Block et al. 2015; Kearns et al. 2018; Müller et al. 2025; Rhymer and Simberloff 1996). The increased knowledge of the evolutionary consequences of hybridisation in combination with the ease with which new genomic data can be generated has revitalised hybridisation as a topic in evolutionary biology. Hybrid zones in particular have commanded considerable interest for the study of hybridisation dynamics and the underlying mechanisms that lead to reproductive isolation between species (Ellegren et al. 2012; Poelstra et al. 2014; Runemark et al. 2018; Schumer et al. 2017; Toews et al. 2016).

Historical introgression events leave distinctive signatures in species´ genomes. Consequently, studies of hybridisation patterns of entire radiations can elucidate the spatio‐temporal frequency of hybridisation. Combining this knowledge with information on environmental and geological dynamics, makes it possible to reveal historical factors that may have triggered hybridisation. Empirical observations and theory support the view that the loss of environmental heterogeneity can increase genetic admixture by decreasing divergent selection and reducing barriers to gene flow (Seehausen et al. 2008). Likewise, climate‐induced range shifts have also been shown to promote hybridisation in several organismal groups (Arce‐Valdés and Sánchez‐Guillén 2022; Canestrelli et al. 2017; Chunco 2014). In birds, introgression has been found to be highest in species occurring in close geographic proximity and in species that occupy areas with more dynamic climate (Singhal et al. 2021). Thus, one can expect introgressive hybridisation to be more frequent in species radiations that occur in unstable regions having repeated changes in environmental heterogeneity (e.g., dynamic island environments), because continuous distributional range changes may lead to recently formed and otherwise allopatric species being brought into secondary contact. Furthermore, introgressive hybridisation may be more frequent for species with high dispersal capacity. Such species can disperse more frequently to areas occupied by closely related newly formed species, which have not undergone sufficient time to become reproductively isolated. However, estimation of introgression patterns in species groups with complex evolutionary histories may be challenging because of the assumptions and concomitant limitations in various tests for introgression (Hibbins and Hahn 2022; Pease and Hahn 2015). Furthermore, it is notoriously difficult to distinguish between competing genomic signals caused by introgression and incomplete lineage sorting (Meng and Kubatko 2009; Pease and Hahn 2013).

The avian family comprising whistlers and their allies (Aves: Pachycephalidae) has an Australo‐Papuan origin (Brady et al. 2022; Jønsson et al. 2011) and includes 69 small to medium‐sized passerine birds (Gill et al. 2024). They generally occur in wooded habitats, particularly rainforests, some species having specific habitat requirements occurring, for example, only in mangrove (del Hoyo et al. 2007). Typical whistlers from the species‐rich genus *Pachycephala* have been particularly successful in colonising a multitude of remote islands across the Indo‐Pacific (Jønsson et al. 2014), which is an environmentally highly dynamic island region. All species of typical whistlers have a similar lifestyle and body shape, but differ to various degrees in size, vocalisations and often in coloration and plumage patterns (del Hoyo et al. 2007). The current knowledge of typical whistlers´ colonisation history of the Indo‐Pacific, their phylogenetic relationships and population structures rests largely on studies based on limited genetic data (Andersen et al. 2014; Jønsson et al. 2010, 2014) or lacking more comprehensive sampling (Brady et al. 2022). Consequently, the extent to which species of typical whistlers hybridise and how this has affected their evolutionary history remains poorly known (but see Joseph et al. 2021). Here, we focus on one monophyletic clade of whistlers to investigate the recurrence of introgressive hybridisation and how this has shaped its evolutionary history. Many taxa within this clade inhabit lowlands in the Sahul region (Australia, New Guinea and surrounding continental islands), in which Pleistocene sea‐level changes have repeatedly altered the configuration and connectivity of landmasses. During Pleistocene ice ages, including the Last Glacial Maximum about 18,000 years ago, sea levels dropped by up to 140 m (Sathiamurthy and Voris 2006; Voris 2000). This led to repeated expansions of landmasses on the Sahul Shelf and corresponding connections among Australia, New Guinea and present‐day continental islands such as Aru and Misool. However, some taxa within this radiation have colonised oceanic and archipelagic islands outside the Sahul region, and these islands have remained isolated during the Pleistocene (Hall 2002, 2012). Recent studies of other groups of birds from the region (Andersen et al. 2021; DeRaad et al. 2023), the high dispersal capacity and the distribution of these birds in an environmentally and geologically dynamic region, together make it likely that high levels of introgressive hybridisation have occurred within this clade. This is supported by incongruence between mitochondrial data (Jønsson et al. 2019), morphology (del Hoyo et al. 2007) and the limited nuclear data available (Brady et al. 2022).

Herein, we address problems such as limited taxon sampling in earlier work and sequence 44 genomes containing individuals from all described taxa (currently 7 species and 21 subspecies are recognised) within this clade (Gill et al. 2024). This allows us to obtain detailed insights into the phylogenomic and hybridisation history of this clade.

## 2 Materials and Methods

### 2.1 Specimen Sampling & Sequencing

**FIGURE 1:**
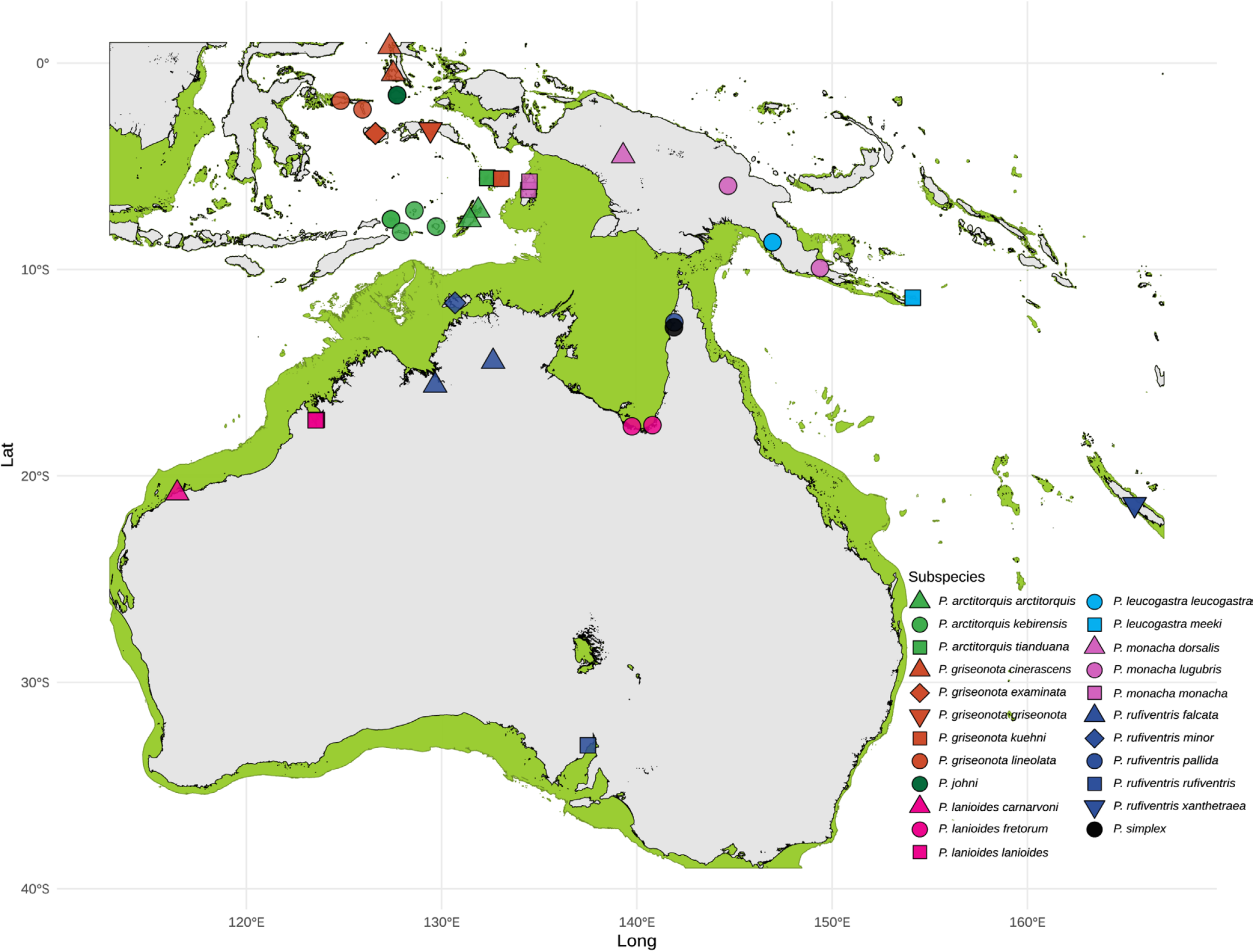
Map showing the study area, sample localities and land connectivity during Pleistocene glacial maxima. The green areas on the map are land exposed during glacial maxima at sea level 140 m below current level. The coloured symbols on the map indicate sample localities for taxa included in the study (see legend). In this study, taxa distributed on the Sahul shelf are classified as Sahul taxa as they had opportunities to get into secondary contact via land bridges during periods of low sea levels. Sahul taxa include all subspecies of *P. lanioides* , *P. rufiventris* , *P. monacha* and *P. leucogastra* , except *P. rufiventris xanthetraea* and *P. leucogastra meeki* . The latter two taxa and all *P. griseonota* , *P. johni* and *P. arctitorquis* subspecies are classified as oceanic/archipelagic taxa as they occupy island that remained disconnected from the Sahul shelf during Pleistocene glacial maxima.

We obtained 11 fresh samples and 32 toepad samples from 21 taxa representing all subspecies of seven species of a monophyletic clade of the Pachycephalidae: *Pachycephala rufiventris* Latham, 1801; *Pachycephala lanioides* Gould, 1840; *Pachycephala griseonota* G. R. Grey, 1862; *Pachycephala johni* Hartert, 1903; *Pachycephala arctitorquis* Sclater, 1883; *Pachycephala monacha* Gray, 1858; and *Pachycephala leucogastra* Salvadori & D'Albertis, 1875. One sample of *Pachycephala simplex* Gould, 1843 was also sequenced to be used as an outgroup for the phylogenetic analyses. Sample localities are shown in Figure 1, and metadata for all samples (such as Study ID, specimen ID, tissue type, sex, coordinates/locality, genome coverage and whether they are classified as continental (Sahul) or oceanic taxa) are listed in Table 1. Genomic DNA from fresh samples was extracted with a KingFisher Duo magnetic particle processor (Thermo Fisher Scientific) using the KingFisher Cell and Tissue DNA Kit. Library preparation (using Illumina TruSeq DNA Library Preparation Kit) and sequencing on Illumina NovaSeq (2 × 150 bp) was performed by SciLifeLab, Stockholm. For detailed descriptions of laboratory procedures for degraded DNA samples from museum specimens see Irestedt et al. (2022) and Meyer and Kircher (2010).

**TABLE 1:** Metadata for all individuals included in this study. These include taxon name, museum voucher number, locality information, tissue type, genome coverage and whether classified as a continental or an oceanic taxon.

Taxon	Voucher	Locality	Tissue type	Year	Coverage	Lat	Long	Classification	ENA accession
*Pachycephala rufiventris minor*	ANWC B48538	Melville Island, NT, Australia	Fresh tissue	1996	23.4	−11.62	130.69	Continental	ERS21338560
*Pachycephala rufiventris falcata*	ANWC B33455	n NT, Australia	Fresh tissue	2002	21.4	−15.62	129.67	Continental	ERS21338561
*Pachycephala rufiventris falcata*	ANWC B33428	n NT, Australia	Fresh tissue	2002	18.1	−14.46	132.64	Continental	ERS21338562
*Pachycephala rufiventris pallida*	ANWC B29820	Cape York, Australia	Fresh tissue	2001	22.1	−12.56	141.92	Continental	ERS21338563
*Pachycephala rufiventris rufiventris*	ANWC B46667	SA, Australia	Fresh tissue	1994	21.4	−33.05	137.49	Continental	ERS21338564
*Pachycephala rufiventris xanthetraea*	NHMUK 1969.41.1179	New Caledonia	Toepad	1880	7.1	−21.38	165.49	Oceanic	ERS21338565
*Pachycephala rufiventris xanthetraea*	NHMUK 88.5.3.309	New Caledonia	Toepad	1880	6.0	−21.38	165.49	Oceanic	ERS21338566
*Pachycephala monacha monacha*	NHMUK 1910.12.28.253	Aru, Indonesia	Toepad	1909	8.5	−6.15	134.45	Continental	ERS21338567
*Pachycephala monacha monacha*	AMNH SKIN 658819	Wokan Island, Aru, Indonesia	Toepad	1900	1.9	−5.75	134.50	Continental	ERS21338568
*Pachycephala monacha lugubris*	ANWC B02443	Kubor Range, Eastern Ranges, New Guinea	Toepad	1963	18.3	−5.94	144.67	Continental	ERS21338569
*Pachycephala monacha lugubris*	ANWC B08239	Milne Bay, SE Peninsula, New Guinea	Toepad	1969	14.8	−9.93	149.38	Continental	ERS21338570
*Pachycephala monacha dorsalis*	AMNH SKIN 341653	Balim River, New Guinea	Toepad	1938	4.0	−4.50	139.30	Continental	ERS21338571
*Pachycephala leucogastra leucogastra*	AMNH 659279	Central Province, SE Peninsula, New Guinea	Toepad	1905	9.0	−8.68	146.95	Continental	ERS21338572
*Pachycephala leucogastra leucogastra*	AMNH 659281	Central Province, SE Peninsula, New Guinea	Toepad	1904	5.9	−8.68	146.95	Continental	ERS21338573
*Pachycephala leucogastra meeki*	AMNH SKIN 659225	Rossel Island, New Guinea	Toepad	1915	4.4	−11.37	154.13	Oceanic	ERS21338574
*Pachycephala leucogastra meeki*	AMNH SKIN 659233	Rossel Island, New Guinea	Toepad	1898	2.3	−11.37	154.13	Oceanic	ERS21338575
*Pachycephala arctitorquis kebirensis*	NRM 552152	Romang Island, Indonesia	Toepad	1902	7.8	−7.57	127.41	Oceanic	ERS21338576
*Pachycephala arctitorquis kebirensis*	NHMUK 1904.7.21.43	Romang Island, Indonesia	Toepad	1902	6.1	−7.57	127.41	Oceanic	ERS21338577
*Pachycephala arctitorquis kebirensis*	AMNH SKIN 295117	Moa Island, Indonesia	Toepad	1902	5.5	−8.17	127.93	Oceanic	ERS21338578
*Pachycephala arctitorquis kebirensis*	AMNH SKIN 658862	Babar Island, Indonesia	Toepad	1905	5.1	−7.92	129.72	Oceanic	ERS21338579
*Pachycephala arctitorquis kebirensis*	AMNH SKIN 658843	Damar Island, Indonesia	Toepad	1898	4.4	−7.14	128.61	Oceanic	ERS21338580
*Pachycephala arctitorquis tianduana*	AMNH SKIN 659275	Tayandu, Indonesia	Toepad	1900	6.2	−5.56	132.34	Oceanic	ERS21338581
*Pachycephala arctitorquis tianduana*	AMNH SKIN 659274	Tayandu, Indonesia	Toepad	1900	8.0	−5.56	132.34	Oceanic	ERS21338582
*Pachycephala arctitorquis arctitorquis*	AMNH SKIN 658886	Larat, Tanimbar, Indonesia	Toepad	1901	3.7	−7.14	131.88	Oceanic	ERS21338583
*Pachycephala arctitorquis arctitorquis*	AMNH SKIN 658896	Yamdena, Tanimbar, Indonesia	Toepad	1901	2.5	−7.60	131.43	Oceanic	ERS21338584
*Pachycephala griseonota lineolata*	AMNH SKIN 658795	Sula Besi, Sula Islands, Indonesia	Toepad	1897	6.3	−2.24	125.95	Oceanic	ERS21338585
*Pachycephala griseonota lineolata*	NHMUK 1934.10.21.108	Taliabu, Sula Islands, Indonesia	Toepad	1934	6.4	−1.82	124.83	Oceanic	ERS21338586
*Pachycephala griseonota cinerascens*	AMNH SKIN 658803	Ternate, N Moluccas, Indonesia	Toepad	1896	7.1	0.79	127.34	Oceanic	ERS21338587
*Pachycephala griseonota cinerascens*	AMNH SKIN 658796	Batjan, N Moluccas, Indonesia	Toepad	1897	6.2	−0.52	127.52	Oceanic	ERS21338588
*Pachycephala griseonota examinata*	NHMUK 1923.9.15.127	Buru, S Moluccas, Indonesia	Toepad	1922	10.3	−3.41	126.61	Oceanic	ERS21338589
*Pachycephala griseonota examinata*	NHMUK 1923.9.15.128	Buru, S Moluccas, Indonesia	Toepad	1922	12.6	−3.41	126.61	Oceanic	ERS21338590
*Pachycephala griseonota griseonota*	NHMUK 1910.12.28.235	Seram, S Moluccas, Indonesia	Toepad	1909	8.5	−3.22	129.43	Oceanic	ERS21338591
*Pachycephala griseonota griseonota*	NHMUK 1910.12.28.236	Seram, S Moluccas, Indonesia	Toepad	1909	8.4	−3.22	129.43	Oceanic	ERS21338592
*Pachycephala griseonota kuehni*	ZMUC 26823	Kai, S Moluccas, Indonesia	Toepad	1880	1.6	−5.60	133.06	Oceanic	ERS21338593
*Pachycephala griseonota kuehni*	NHMUK 1900.11.1.10	Kai, S Moluccas, Indonesia	Toepad	1898	11.0	−5.60	133.06	Oceanic	ERS21338594
*Pachycephala johni*	AMNH SKIN 658812	Obi, N Moluccas, Indonesia	Toepad	1902	4.4	−1.55	127.72	Oceanic	ERS21338595
*Pachycephala johni*	AMNH SKIN 658813	Obi, N Moluccas, Indonesia	Toepad	1902	5.5	−1.55	127.72	Oceanic	ERS21338596
*Pachycephala lanioides carnarvoni*	ANWC B33208	30 km W of Karratha, WA, Australia	Fresh tissue	2002	20.2	−20.84	116.46	Continental	ERS21338597
*Pachycephala lanioides lanioides*	ANWC B50489	King Sound, NW of Derby, WA, Australia	Fresh tissue	2004	19.1	−17.29	123.61	Continental	ERS21338598
*Pachycephala lanioides lanioides*	ANWC B50512	Mary Island South, WA, Australia	Fresh tissue	2004	27.3	−17.31	123.55	Continental	ERS21338599
*Pachycephala lanioides fretorum*	ANWC B29494	Qld, Australia	Fresh tissue	2001	21.0	−17.54	140.80	Continental	ERS21338600
*Pachycephala lanioides fretorum*	ANWC B32677	Qld, Australia	Fresh tissue	2001	16.8	−17.60	139.75	Continental	ERS21338601
*Pachycephala simplex* (*outgroup*)	ANWC B29879	Qld, Australia	Fresh tissue	2001	21.6	−12.8	141.9	Continental	ERS21338602

### 2.2 Read Cleaning and Mapping

The sequenced reads were cleaned using the Nextflow‐based nf‐polish (https://github.com/MozesBlom/nf‐polish). In short, the pipeline performs deduplication, trimming of adapters, removing low‐quality and low‐complexity reads and merging overlapping read pairs. The cleaned reads were then mapped to a de novo assembly of *Pachycephala schlegelii* Schlegel, 1871 (GenBank assembly accession GCA_040366215.1) using nf‐μmap (https://github.com/IngoMue/nf‐umap). Through this pipeline, we applied bwa‐mem2 as the mapping algorithm (v.2.2.1, (Vasimuddin et al. 2019)). Additionally, we performed mapping quality control through qualimap *2* (v.2.2.2d, (Okonechnikov et al. 2016)) and used DamageProfiler (v.0.4.9, (Neukamm et al. 2021)) to check for damage patterns that are typical for historical DNA.

### 2.3 SNP Calling

As the reference genome consisted of more than 24,000 scaffolds, we subset the genome for downstream analysis to the largest 183 scaffolds which together covered 90% of the genome. Within this subset, we identified scaffolds that are linked to either sex chromosome by aligning each scaffold against chromosome‐level assemblies from *Lycocorax pyrrhopterus obiensis* Bernstein, 1865 (GenBank assembly accession GCA_014706295.1) and *Corvus cornix* Linnaeus, 1758 (RefSeq assembly accession GCF_000738735.6) using minimap2 (v. 2.24, (Li 2018)). If a scaffold aligned with at least 75% of its length against either the Z or W chromosome, it was considered sex chromosome linked; if less than 25% of a scaffold aligned to a sex chromosome, it was considered autosomal. Scaffolds which had 25%–75% of their length aligned against a sex chromosome were discarded from the analysis as we could not identify their origin with high confidence. Sex‐linked scaffolds that were identified this way covered 72,919,367 bp (corresponding to 97.89% of the *L. pyrrhopterus* Z chromosome), and the remaining autosomal scaffolds covered 941,758,833 bp (82.38% of the *P. schlegelii* assembly). Using these subsets, we performed joint variant calling using freebayes (v. 1.3.0, (Garrison and Marth 2012)) on all individuals for (1) only autosomal scaffolds and (2) Z‐linked scaffolds.

### 2.4 Phylogenetic Inference and Mitochondrial Dating

To investigate the evolutionary history of *Pachycephala* species, we employed two complementary approaches. First, we inferred a primary phylogenetic tree by concatenating 3145 autosomal partitions, each 10,000 base pairs in length. Second, we inferred individual trees for each partition independently. These partitions corresponded to orthologous regions of the *Pachycephala* genomes, selected based on 100 kb windows with read coverage among all species. For both approaches, we conducted model selection for each partition using ModelFinder (Kalyaanamoorthy et al. 2017), selecting the optimal model based on the Bayesian Information Criterion (BIC) (‐MFP option in IQ‐TREE v. 2.2.2.6 (Minh et al. 2020)). In the first approach, we applied the best‐fitting models to the corresponding partitions in IQ‐TREE, with the concatenated sequence data serving as input. In the second approach, we inferred a phylogenetic tree for each partition independently, using its best‐fitting model. Subsequently, phylogenies were inferred using a Maximum Likelihood (ML) framework within IQ‐TREE (v.2.2.2.6 (Minh et al. 2020)). For tree reconstruction, we utilised the edge‐linked partition model (‐spp option) (Chernomor et al. 2016), which allows each gene to have its own evolutionary rate. Node support for key relationships was assessed using Ultra‐Fast Bootstrap (Hoang et al. 2018) and SH‐aLRT (Anisimova et al. 2011) analyses (‐bb 1000 and ‐alrt 1000). To minimise the risk of overestimating branch support due to potential model violations, we incorporated the ‐‐bnni option. With this IQ‐TREE setting, UFBoot enhances each bootstrap tree by performing a hill‐climbing nearest neighbour interchange (NNI) search, directly utilising the corresponding bootstrap alignment. To further investigate the concordance of gene trees with the species tree, we conducted a window and site concordance factor (wCF and sCF) also implemented in IQ‐TREE (Mo et al. 2023). The concordance factors were calculated for both gene trees (‐‐gcf) and site trees (‐‐scf), with 100 replicates for site concordance factors. The same phylogenetic reconstruction approach was applied to infer a phylogeny using partitions from the Z chromosome. For this analysis, 270 partitions of 10,000 base pairs each were concatenated to build the tree.

We also constructed autosomal and Z chromosome densiTrees from the R package phangorn (Schliep 2011) to illustrate the topological differences of the 3145 and the 270 phylogenetic trees obtained from the two data sets, respectively. The density and thickness of the superimposed trees visually convey the consensus and variability among them, highlighting the most common branches in the dataset.

The mitochondrial scaffold was unnamed in the assembly of the *P. schlegelii* genome used for mapping of the cleaned reads. In order to identify the correct scaffold corresponding to the mitochondrial genome, we used blastn (Altschul et al. 1990) to search for scaffolds between 10,000 bp and 20,000 bp (the expected size is around 17,000 bp for birds) present in the reference assembly, extracting them with seqtk (https://github.com/lh3/seqtk). Since read depth is expected to be higher in mitochondria compared to nuclear DNA due to the higher copy number of mitochondria, we only considered scaffolds with a read depth higher than 100× based on the sample *P. monacha lugubris* B02443 (the museum sample with the highest read depth). Through this method, we identified scaffold 107,700 as the mitochondrial scaffold due to a high query match (99%) and high identity match (92%) with mitochondrial genomes of *Pachycephala* species present in the blast database. We then extracted scaffold 107,700 from the bam files of all samples with samtools view seqtk (Danecek et al. 2021). These scaffolds were aligned with MAFFT (v.7.407; (Katoh et al. 2002)) using the parameter settings ‐‐maxiterate of 1000 and ‐‐globalpairs. A phylogeny was built using RaxML‐NG (v.1.1.0; (Stamatakis 2014)) with the MAFFT alignment using the GTR + G model and 100 bootstrap iterations.

We used the same mitochondrial alignment as for the phylogenetic tree to estimate divergence times between our taxa through BEAST2 (v. 2.7.4, (Bouckaert et al. 2019)). The underlying substitution model was GTR + G + I (as suggested by ModelTest‐NG (Darriba et al. 2020)) with eight gamma rate categories, the tree model was the Coalescent Bayesian Skyline (Drummond et al. 2005) and we applied a clock rate of 0.0205 through the relaxed log normal clock model (Drummond et al. 2006). This clock rate was calculated as the average rate throughout mitochondrial regions estimated in the supplement to (Lerner et al. 2011). BEAST2 was run through an MCMC chain of 10^8^ steps, after burning‐in 10^7^ steps. Every 1000 steps were recorded in a log file. Effective sample sizes (ESS) were confirmed sufficiently high (lowest value: 384, most ESS well above 1000) with Tracer (v. 1.7.2, (Rambaut et al. 2018)) and the target tree was obtained through TreeAnnotator (Drummond and Rambaut 2007) using default settings except for median heights to determine node heights. The final dated phylogeny was visualised using FigTree (v 1.4.4, (Rambaut 2018)).

### 2.5 Population Structure and Tests of Introgression

To prevent biased estimates of population structure and introgression caused by the influence of adaptive, physically linked regions, we first filtered our SNP dataset for linkage disequilibrium using PLINK with the parameter ‐‐indep‐pairwise 50 10 0.1. We then conducted a genome‐wide principal component analysis (PCA) on all individual genomes using the software emu (Meisner et al. 2021). We calculated the first 10 principal components based on autosomal biallelic SNPs, using only the sites with less than 10% missing genotypes and applying a minor allele frequency filter of 5%. Admixture components for each genome were estimated using the software ADMIXTURE (v.1.3.0, (Alexander et al. 2009)). We calculated admixture components for *K* = 2 to *K* = 9 clusters, using all autosomal biallelic sites with < 10% missing genotypes.

To investigate patterns of gene flow, we calculated the excess of derived allele sharing between all (sub)species pairs using the ABBA‐BABA test (Durand et al. 2011). This was applied to all possible pairwise combinations where (sub)species P1 and P2 shared more derived alleles with each other than either did with P3, ensuring that P1 and P2 are genetic sister species relative to P3. All calculations were performed using the software Dsuite (Malinsky et al. 2021).

In order to further investigate introgression patterns, TreeMix (v.1.13, (Pickrell and Pritchard 2012)) was run on allele frequencies estimated from the (sub)species groupings. We first converted the autosomal biallelic VCF genotype calls into TreeMix format using custom Python scripts, counting the number of ancestral and derived alleles relative to the reference for each (sub)species group. A maximum likelihood (ML) tree was then constructed using TreeMix, accounting for linkage disequilibrium (LD) by grouping sites into blocks of 1000 SNPs (‐k 1000). After adding all populations to the tree, a round of rearrangements was performed (‐global). Following the construction of the ML tree, migration events were added (‐m) for each value of ‘m’ (1–3). The inferred ML trees were visualised using the built‐in R plotting functions in TreeMix.

We also investigated gene flow between populations using Fbranch statistics implemented with Dsuite (Malinsky et al. 2021). We included one sample per subspecies and ran Dsuite for Fbranch statistics with the freebayes‐produced VCF for all possible trio‐combinations using a species tree based on the phylogenetic reconstruction of the autosomes as a guide tree.

To compute the uncorrected pairwise genetic distance (p‐distance) between genomes, we utilised the VCF file previously used for the TreeMix analysis. The file was parsed using the cyvcf2 library in Python (Pedersen and Quinlan 2017). Each sample's genotype data was extracted from the VCF file and a genotype matrix was generated. For each pair of samples, the genetic distance was calculated as the number of variant sites where the genotype differs between two samples. This was achieved by comparing the alleles for each pair across all variant positions. The pairwise genetic p‐distance were then plotted in a boxplot manner using the ggplot2 package implemented in R (Wickham 2016).

## 3 Results

### 3.1 Genome Coverage, Phylogenetic Results and Mitonuclear Discordance

After read cleaning and mapping, the genome coverages ranged from 1.6× to 27.3× when mapped to the *P. schlegelii* reference genome (Table 1). The genomic data generated from fresh tissue samples had on average higher coverage (mean: 21.1×) yet the genomic data generated from museum samples had coverage ranging from 2.3 to 18.3× (mean: 6.8×) except for two samples ( *P. monacha monacha* AMNH SKIN 658819 and *P. griseonota kuehni* ZMUC 26823) that had a coverage below 2×. These two samples were discarded from downstream analyses. All raw reads generated for this study have been deposited at the European Nucleotide Archive, accession number PRJEB81482 (individual accession number are given in Table 1).

**FIGURE 2:**
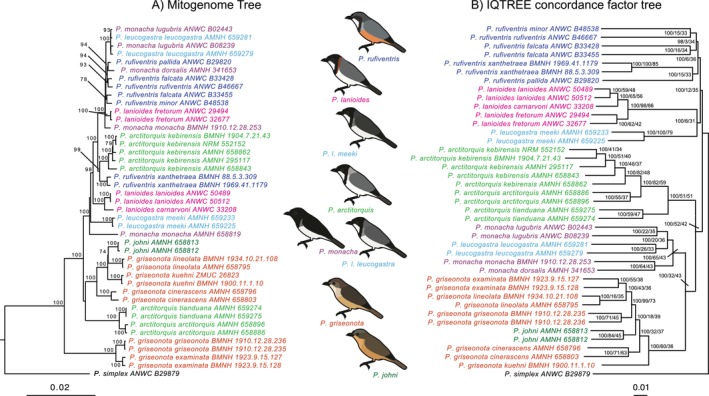
Two phylogenetic trees depicting the significantly different phylogenetic signals obtained from the mitochondria and the nuclear genomes. The mitochondrial tree (A) is inferred from almost complete mitochondria using RaxML‐NG (bootstrap values are provided at nodes), while the nuclear tree (B) is inferred from 3145 autosomal nuclear regions with IQ‐TREE (support values at nodes are provided as follow UFBoot/wCF/sCF). In the mitochondrial tree, none of the traditionally recognised species (except for the monotypic *P. johni* ) form monophyletic clades, all Sahul taxa are genetically very similar to each other (except two *P. lanioides* subspecies) and densely packed, whereas oceanic/archipelagic taxa have mitochondria that are more divergent from each other. In contrast, traditionally recognised species are generally forming monophyletic clades in the nuclear tree (with the exception of *P. leucogastra* and that *P. johni* is nested within the *P. griseonota* clade), and the nuclear tree is thus more in line with relationships expected from morphology and distributions. However, the relationships among major clades in the nuclear tree are generally very poorly supported as evident from very low site and window concordance factors.

While most nodes in the mitochondrial tree (Figure 2A) are well supported, the autosomal nuclear tree contains many clades that are surprisingly poorly resolved, despite drawing on whole genomic data (Figure 2B). The topology of the mitochondrial tree deviates extensively from the nuclear tree and does not support monophyly of traditionally recognised species. The nuclear tree, on the other hand, generally supports the current taxonomy with the exception of *P. leucogastra* , which is paraphyletic. *P. leucogastra leucogastra* is nested within *P. monacha* and *P. leucogastra meeki* is sister to *P. lanioides* . In the nuclear tree, *P. rufiventris* , *P. lanioides* and *P. leucogastra meeki* form a sister clade to all other *Pachycephala* taxa in this study, but in the mitochondrial tree, members of *P. rufiventris* are paraphyletic with respect to various Sahul taxa from the other clade recovered from nuclear data ( *P. monacha* , *P. leucogastra leucogastra* ). This is indicative of extensive recent introgression of mitochondrial genomes between Sahul species. There is also phylogenetic incongruence between the nuclear and the mitochondrial tree for oceanic/archipelagic taxa ( *P. griseonota* , *P. arctitorquis P. leucogastra meeki* and *P. rufiventris xanthetraea* ), but in the mitochondrial tree it is less extensive and the incongruences concern deeper nodes.

**FIGURE 3:**
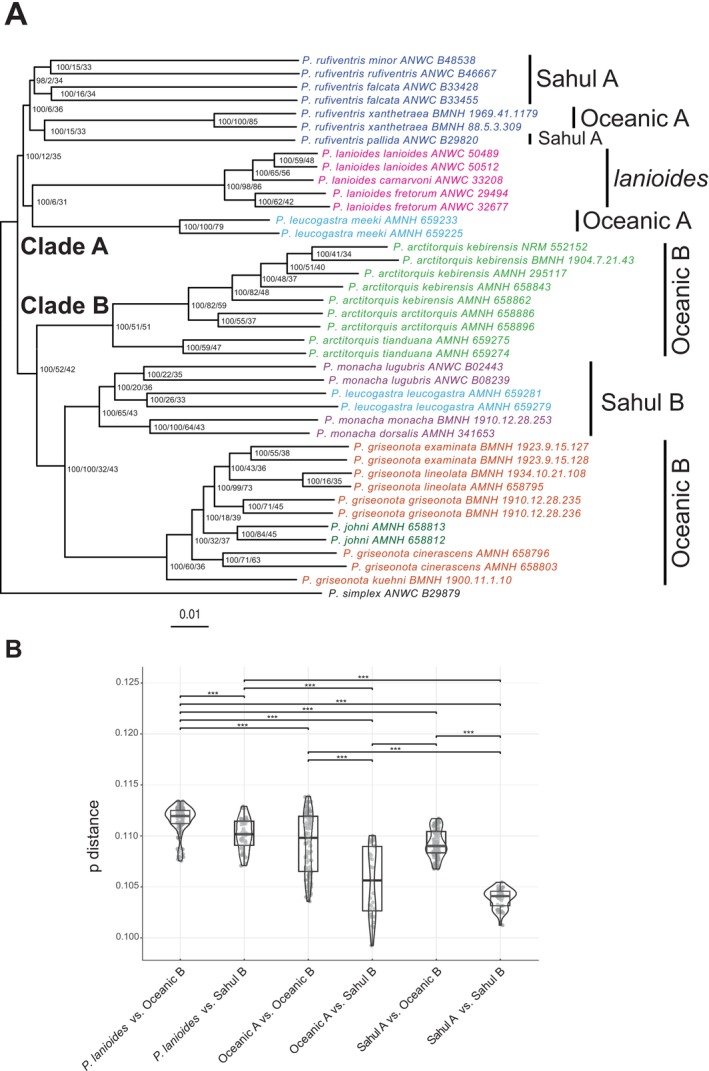
Comparisons of genetic p‐distances between major groups across the two main lineages in the autosomal phylogenetic tree. (A) The autosomal phylogenetic tree where clades for which genetic p‐distance has been calculated are shown to the right (support values at nodes are provided as follow UFBoot/wCF/sCF). (B) Violin plots showing the calculated p‐distances for the groups compared. Comparisons that differ significantly are indicated with ***p < 0.001. The results show that the genetic distances between groups in the two main clades are significantly shortest between Sahul taxa.

In the autosomal nuclear tree (Figure 2B), the deeper nodes are poorly supported (low site and window concordance factors). This is particularly true for the nodes connecting the branch leading to the monophyletic origin of *P. rufiventris* , *P. lanioides* and *P. leucogastra meeki* (clade A in Figure 3) and the node leading to the monophyletic origin of *P. rufiventris* (including internal nodes within this clade). Furthermore, several basal nodes for Sahul taxa within the other main clade (clade B in Figure 3) are poorly supported. The basal patterns in the nuclear tree where different genetic regions support competing relationships are striking. They are congruent either with an early rapid radiation, which has made ancestral gene regions fail to coalesce to the same topology (ILS—incomplete linage sorting) or with extensive introgressive hybridisation having introduced competing phylogenetic signals. The densiTree results (Figures S1 and S2) clearly show the extensive competing phylogenetic signals in the autosomes and the Z chromosome. The introgression history of the Z chromosome is not clearly different from that of the autosomes, even though the Z chromosome is expected to be more shielded from introgression in birds (Backström et al. 2010; Blom et al. 2024).

The Z chromosome tree (Figure S3) similarly has very low support (site and window concordance factors) for nodes at basal positions. With that caveat, it is largely congruent with the autosomal tree. An exception is the position of *P. lanioides* . Instead of being part of a larger clade comprising *P. rufiventris* , *P. lanioides* and *P. leucogastra meeki* , it is sister to all taxa in the other main autosomal clade.

The dated mitochondrial tree (Figure S4) suggests that the focal clade started to diverge around 800 kya and further supports young (< 200 kya) mitochondrial divergences between *P. rufiventris* (except *P. rufiventris xanthetraea* ), *P. monacha* , *P. leucogastra* and *P. lanioides fretorum* . This supports that mitochondrial capture across the latter taxa has occurred in recent times. Due to the extensive signature of mitochondrial capture across taxa in the focal clade, the mitochondrial dating should not be interpreted as absolute divergence times in the study.

### 3.2 Population Structures, Genetic Distances Between Main Clades and Signatures of Hybridisation

**FIGURE 4:**
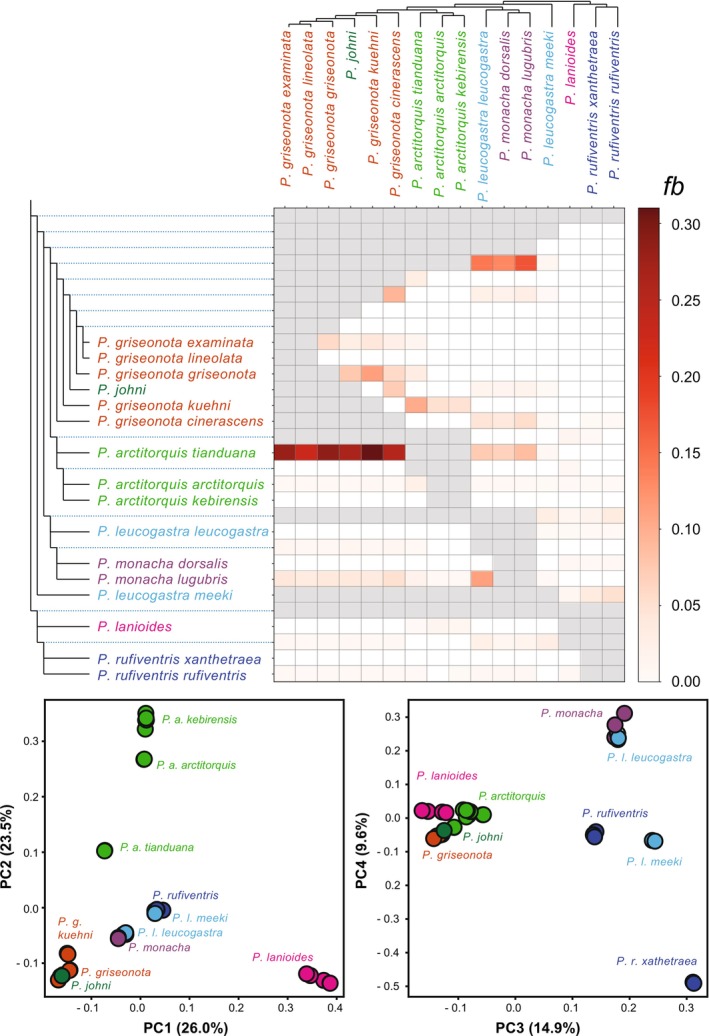
Fbranch statistic and Principal component analysis (PCA). Fbranch statistic (above) calculated using Dsuite indicating historical gene flow. Particular elevated signatures of gene flow exist between *P. arctitorquis tianduana* and species within the *P. griseonota* / *P. johni* complex, as well as between *P. monacha* / *P. leucogastra leucogastra* and ancestral lineages leading to the *P. griseonota* / *P. johni* complex. Principal component analysis, PCA (below), based on genotype likelihoods (autosomes) where the PC1 to PC4 axes together explain 74% of the genic variation in the data set.

On the first four Principal Component (PC) axes of the autosomal data (Figure 4) the groupings of taxa are largely congruent with the autosomal phylogenetic results. For example, *P. leucogastra leucogastra* clusters with *P. monacha* . *P. johni* clusters with *P. griseonota* on all four PC axes, whereas *P. leucogastra meeki* and *P. rufiventris xanthetraea* cluster tightly with other members of *P. rufiventris* on PC axes 1 and 2 but are well separated from each other and other members of *P. rufiventris* on PC axes 3 and 4. The intermediate position of *P. arctitorquis tianduana* between other members of *P. arctitorquis* and *P. griseonota* / *P. johni* on PC axes 1 and 2 is particularly notable. Similarly, the clustering of Sahul *P. rufiventris* (except oceanic *P. rufiventris xanthetraea* ) relatively close to the Sahul *P. monacha* / *P. leucogastra leucogastra* cluster across all four PC axes. This is striking, as these two groups belong to separate main clades in the autosomal phylogenetic tree (clades A and B, respectively, in Figure 3).

These results suggest that *P. arctitorquis tianduana* is a hybrid population with mixed ancestry from both *P. arctitorquis* and *P. griseonota* . The results also indicate that the two Sahul groups, *P. rufiventris* (excluding *P. rufiventris xanthetraea* ) and *P. monacha* / *P. leucogastra leucogastra* , are genetically more similar to each other than to other members across these two main clades. This pattern aligns with uncorrected pairwise genetic distances (autosomal data), which show that individuals classified as Sahul taxa (the Sahul A and Sahul B clades) have significantly lower genetic distances between them than between individuals classified as oceanic taxa (the Oceanic A clade and the Oceanic B clade) (Figure 3). Additionally, it is notable that genetic distances between Sahul *P. lanioides* and taxa belonging to both the Sahul B clade and the Oceanic B clade are comparatively high (Figure 3).

The admixture results (Figure S5) are complex because taxa show signs of admixture depending on K values. Some patterns are conspicuous and largely congruent with the PCA and the uncorrected genetic distances. For example, at *K* = 3 and 4, Sahul species (excluding *P. lanioides* ) cluster together but include ancestral components from other clusters that agree with the phylogenetic results. For example, all *P. rufiventris* samples (except *P. rufiventris xanthetraea* ) have components from *P. lanioides* at both *K* = 3 and 4; *P. monacha* and *P. leucogastra leucogastra* have components from *P. griseonota* /*johni* at *K* = 4, while oceanic *P. rufiventris xanthetraea* and *P. leucogastra meeki* lack components from any of these groups at both *K* = 3 and 4. Additionally, *P. arctitorquis tianduana* is supported as a hybrid population between the two species *P. arctitorquis* and *P. griseonota* at almost all *K* values. The *K* value with the lowest cross‐validation errors is *K* = 4 (Figure S5), but differences in cross‐validation errors between *K* values are minor (with a tendency that the cross‐validation values increase with increased *K* values), and *K* = 4 aligns poorly with current classifications and groupings in the PCA.

The Patterson's D‐statistic (ABBA‐BABA) heatmap (Figure S6) is complex, possibly due to the extensive amount of hybridisation within the species complex, which may result in extensive carry‐over effects (Jensen et al. 2024). Aligning with our other lines of evidence, *P. arctitorquis tianduana* shows high signatures of allele sharing with the *P. griseonota* / *P. johni* clade, and *P. lanioides* shows comparatively low signatures of allele sharing with other taxa. We also observed signatures of allele sharing between the *P. griseonota* / *P. johni* and *P. leucogastra leucogastra* / *P. monacha* clades, which are not evident from all other analyses in this study but supported by the Fbranch statistic (below) and the admixture analysis at *K* = 4. It is more difficult to explain why we recover only a very slight signature of allele sharing between Sahul taxa ( *P. rufiventris* and *P. monacha* / *P. leucogastra leucogastra* ), despite their placement in the PCA and the uncorrected pairwise genetic distances suggesting that they are disproportionately closely related when accounting for phylogenetic relationships. However, D‐statistics are only able to detect asymmetric introgression (Durand et al. 2011), for example, the ABBA‐BABA test will not detect allele sharing under conditions of reciprocal gene flow between two lineages. This presents a challenge in the current data set, as the densiTrees results (Figures S1 and S2) indicate that gene flow between species/lineages often is strong in multiple directions. Our claim that the D‐statistic (ABBA‐BABA) is affected by very complex hybridisation histories, which ultimately result in somewhat skewed estimates, is further supported by observations concerning *P. griseonota kuehni* . Within the *P. griseonota* / *P. johni* clade, it is the taxon that shows the least evidence of allele sharing with *P. leucogastra leucogastra* / *P. monacha* , despite being geographically closest to them. Furthermore, *P. griseonota kuehni* exhibits the least allele sharing of all *P. griseonota* /*johni* taxa with *P. arctitorquis tianduana* , which contradicts all our other analyses. These analyses consistently suggest that *P. arctitorquis tianduana* is of hybrid origin, having received genetic material from *P. griseonota kuehni* .

The calculated Fbranch statistic results (Figure 4) are overall similar to those observed in Patterson's D‐statistic (ABBA‐BABA) heatmap. However, compared to Patterson's D‐statistic, the Fbranch statistic is specifically designed to handle complex scenarios involving multiple introgression events across a phylogeny (Malinsky et al. 2021). In line with this, the Fbranch statistic appears to be less affected by carry‐over effects in this data set. For example, the Fbranch statistic suggests that *P. griseonota kuehni* exhibits the highest level of allele sharing with *P. arctitorquis* among all *P. griseonota* /*johni* taxa, which aligns with both the admixture and PCA results. However, even the Fbranch statistic detects only a very faint signature of allele sharing between Sahul taxa ( *P. rufiventris* and *P. monacha* / *P. leucogastra leucogastra* ), despite other lines of evidence in this study suggesting that introgression among Sahul taxa has been among the most extensive in the entire data set. The Fbranch also finds elevated signatures of gene flow between the *P. leucogastra leucogastra* / *P. monacha* clade and the ancestor to the *P. griseonota* clade. Less elevated signatures of gene flow also occur between many other taxa, but most of these are between closely related taxa that occur in close geographical proximity.

Our analysis in TreeMix (allowing for one gene flow event) (Figure S7) also suggests that gene flow between *P. griseonota kuehni* and *P. arctitorquis tianduana* is the most dominant. When allowing for multiple gene flow events, it is still only gene flow between the *P. griseonota and P. arctitorquis* clades that is supported, likely driven by the high hybrid content found in *P. arctitorquis tianduana* .

## 4 Discussion

In young species radiations, the frequency of hybridisation between sympatric lineages tends to be high and many incipient/young species are therefore likely evolutionary short lived in nature (Rosenblum et al. 2012). Increased access to genomic data has shown that introgressive hybridisation, that is, exchange of genetic material between species, is ubiquitous in nature (Mallet et al. 2016; Taylor and Larson 2019), and several cases of lineage fusions have been detected (Behm et al. 2010; Block et al. 2015; Kearns et al. 2018; Taylor et al. 2006). Consequently, studies on hybridisation are important for our understanding of speciation processes and the evolution of biodiversity. The literature is replete with examples of the mechanisms that limit or promote hybridisation across species pairs (e.g., Grant and Grant 1997; Qvarnström et al. 2023), but studies on how environmental and demographic factors affect hybridisation or incomplete lineage sorting across entire species radiations have largely been neglected (but see, e.g., DeRaad et al. 2023; Seehausen et al. 2008). Our genomic data for seven species (21 taxa) of whistlers reveal a pattern where taxa that occur in geographical proximity in environmentally unstable regions show particularly strong signatures of introgression. Our analyses further support that gene flow between young species can be extensive under certain conditions (e.g., for species with high dispersal capacity or in dynamic environments) despite substantial differences in plumages and vocalisation which would be expected to act as barriers to gene flow. However, substantial differences in plumages and vocalisations may be preserved despite extensive ongoing flow (Toews et al. 2016).

### 4.1 Contradictory Patterns of Introgression and ILS

The introgression patterns in the focal whistler radiation are complex and difficult to fully disentangle as the results of different analyses are sometimes contradictory. A detailed investigation of the mechanisms underlying the observed inconsistent introgression patterns is beyond the scope of this study. However, as all analyses conducted in this study have their own assumptions and consequently concomitant limitations, it is not unexpected that the results are sometimes contradictory. For example, D‐statistics were developed to estimate asymmetric introgression by estimating excess of allele sharing (Durand et al. 2011). Consequently, this method has limitations if gene flow between two or more taxa/lineages is approximately equally extensive in all directions. That this might cause problems in our data set is indicated by the densiTree analyses (Figures S1 and S2), which suggest that gene flow between taxa/lineages often occurs in multiple directions. This may explain why the D‐statistic and Fbranch statistic only detect a very faint signal of introgression between the two Sahul groups ( *P. rufiventris* excluding *P. rufiventris xanthetraea* and *P. monacha* / *P. leucogastra leucogastra* ), despite other evidence showing that introgression between these two lineages is among the most extensive in the entire data set (uncorrected pairwise genetic distances, the extensive mitonuclear phylogenetic incongruence, and grouping of these taxa at *K* = 3 and *K* = 4). The challenge of distinguishing between competing genomic signals caused by introgression or incomplete lineage sorting (ILS) (Meng and Kubatko 2009; Pease and Hahn 2013) further complicates a detailed interpretation. It is highly likely that ILS has also contributed to the observed pattern of competing phylogenetic signals in this system. However, despite the difficulties of distinguishing whether genomic patterns are caused by ILS or inherent limitations in the methods, some general patterns are conspicuous and consistent with rampant introgressive hybridisation. Some of the strongest evidence for hybridisation in this data set stems from several sources: the extensive phylogenetic incongruence between the mitochondrial and the nuclear trees (Figure 2), extensive gene tree incongruences (Figures S1 and S2), the significant differences in uncorrected pairwise genetic distances between phylogenetically equally distant clades (Figure 3), and TreeMix, admixture and D‐statistics.

### 4.2 Strong Signatures of Introgression in Taxa Occupying Environmentally Unstable Regions

Geographical proximity is one factor that has increased introgression. This is indicated by the derived allele sharing between *P. griseonota* / *P. johni* and *P. leucogastra leucogastra* / *P. monacha* in the ABBA‐BABA test (Figure S6). However, a very prominent pattern in our data set is that taxa ( *P. monacha* and *P. leucogastra leucogastra* and most subspecies of *P. rufiventris*) inhabiting environments in the geologically unstable Sahul region beyond the Australian continent (Figure 1) tend to have nuclear genomes that are disproportionately genetically similar to each other compared to taxa occupying remote oceanic/archipelagic islands ( *P. griseonota* , *P. arctitorquis* , *P. rufiventris xantheraea* and *P. leucogastra meeki* ). This pattern is significantly supported when uncorrected genetic distances between Sahul taxa in the two clades are compared with the corresponding distances for oceanic/archipelagic taxa as well as for other taxon comparisons (Figure 3) and it is also evident in the PCA (Figure 4) where Sahul species are relatively closely aligned (regardless of phylogenetic relationship). We interpret the lack of a strong introgression signal between the two Sahul clades in both the D‐statistics (Figure S6) and Fbranch statistic (Figure 4) as a consequence of largely symmetric gene flow, that is, gene flow of similar intensity in both directions.

The fact that Sahul taxa are genetically more similar to each other than oceanic taxa (Figure 3), after controlling for phylogenetic relationships, is congruent with geological and environmental instability in the Sahul region, specifically Pleistocene sea‐level fluctuations, having been a primary driver of repeated secondary contact between Sahul species ( *P. rufiventris* , *P. monacha* and *P. leucogastra leucogastra* ). This, in turn, has led to significant introgressive hybridisation and some homogenisation of the nuclear genomes of Sahul taxa. In contrast, taxa on oceanic/archipelagic islands with higher levels of isolation have nuclear genomes that tend to be less introgressed. This scenario is also supported by the mitochondrial tree where most individuals from taxa inhabiting the Sahul region ( *P. monacha* and *P. leucogastra leucogastra* and most subspecies of *P. rufiventris* and *P. lanioides fretorum* ) form a robustly supported clade having short branch lengths and in which currently recognised species are non‐monophyletic (Figure 2). The low mitochondrial divergence in this clade, reflected in the clade essentially forming a polytomy (Figure 2; Figure S4), suggests that extensive mitochondrial introgression has occurred during recent times of low sea levels. In contrast, species inhabiting isolated oceanic/archipelagic islands tend to have mitochondrial sequences that are more divergent from each other. This is also the case for taxa (at the subspecies level) that occupy oceanic/archipelagic islands within the same archipelago ( *P. griseonota* and *P. arctitorquis* ).

However, one Sahul species, the white‐breasted whistler ( *P. lanioides* ), does not follow this general pattern. Its relatively isolated position in the PCA (Figure 4), as well as its markedly greater uncorrected genetic distances to Sahul taxa in the other main clade (Figure 3), suggests clear differentiation without extensive introgressive hybridisation. This sets it apart from other Sahul taxa. We suggest that this species' specialisation to dense mangroves along northern Australia has enhanced its ecological isolation and resulted in selection against hybridisation. On the other hand, two individuals of *P. lanioides* have mitochondria (Figure 2) from the same haplogroup as most other Sahul species. This indicates that this taxon has not been completely protected from introgressive hybridisation.

As smaller population sizes on islands could lead to faster fixation of novel genetic variation (Baum et al. 2017), one may argue that observed differences in genomic divergences between Sahul taxa and oceanic/archipelagic island taxa are the results of differences in population sizes and genetic drift, rather than in the extent of nuclear introgression. However, this would not predict genomic patterns of divergence in *P. lanioides* to deviate from those observed in other Sahul taxa. The observed mitochondrial introgression pattern (Figure 2) is direct evidence that Sahul taxa have exchanged genetic material more recently compared to oceanic/archipelagic island taxa in this study.

### 4.3 Hybrid Origin of the Tayandu Islands Wallacean Whistler

Although taxa on relatively isolated oceanic islands have been less exposed to hybridisation, the genomes of some oceanic/archipelagic taxa do show signatures of hybridisation. For example, this is evident from significant incongruences between the phylogenetic positions of several oceanic/archipelagic island taxa in the mitochondrial and in the nuclear tree (Figure 2). That whistlers on oceanic/archipelagic islands occasionally come into secondary contact and have opportunities to hybridise is not surprising. The present distribution of whistlers (del Hoyo et al. 2007) demonstrates that they are capable of dispersal across open water, which has led them to colonise all significant island landmasses in the Indo‐Pacific, even reaching Samoa in the east.

The Tayandu Islands population of Wallacean whistlers ( *P. arctitorquis tianduana* ) is a striking example that shows the strongest signature of hybridisation in the entire data set in almost all analyses. Our results suggest that it is descended from hybridisation between *P. griseonota* and *P. arctitorquis* , having close to equal genomic contributions from those species (corroborated by its intermediate position between these two species in the PCA (Figure 4) and from its admixed proportions of close to 50% at most *K* values in the Admixture analyses (Figure S5)) The TreeMix analyses (Figure S7) suggest gene flow from *P. griseonota kuehni* into *P. arctitorquis tianduana* . *P. griseonota kuehni* inhabits the Kai Islands, which are located only about 30 km from the Tayandu Islands (inhabited by *P. arctitorquis tianduana* ) surrounded by shallow waters in the Banda Sea. Thus, these two populations from two different species have had ample opportunity to come into secondary contact, either when sea levels were low or by short‐distance dispersal. While *P. arctitorquis tianduana* is clearly a close to 50/50% hybrid population between *P. griseonota* and *P. arctitorquis* , its precise evolutionary origin is unclear and two hypotheses may be offered. One is that it formed through classical processes of homoploid hybrid speciation (Mallet 2007) in which a single pair or few individuals from two species interbred followed by no backcrossing to either parental form. Alternatively, its hybrid origin may be a consequence of successive introgression whereby gene flow from *P. griseonota kuehni* into *P. arctitorquis tianduana* has gradually built up an intermediate genetic content in the present *P. arctitorquis tianduana* population. In birds, several species are suggested to have had a hybrid origin (Ottenburghs 2023), but as it is difficult to study evolutionary processes of hybrid speciation in nature (but see Lamichhaney et al. (2018)), it remains uncertain whether *P. arctitorquis tianduana* entirely fulfils the criteria for being a classical homoploid hybrid species (Runemark et al. 2018; Schumer et al. 2014). However, its intermediate gene content and its isolated distribution on the Tayandu Islands makes *P. arctitorquis tianduana* an excellent candidate for testing the hypothesis of a homoploid hybrid origin, a hypothesis that may be resolved with additional sampling and analysis of haplotype block sizes derived from each parental species.

### 4.4 Taxonomic Implications

The current taxonomy of the whistler radiation does not reflect our phylogenetic results (Figure 2) or the clustering patterns observed in the PCA (Figure 4). While a revision of the classification may be necessary, it is complicated by the challenge of representing a largely reticulate evolutionary history rather than a strictly bifurcating tree‐like structure.

Furthermore, as our study shows that young avian species with distinct plumage differences (particularly in males) are able to hybridise extensively (and perhaps even form ‘hybrid species’ as in the case of the Wallacean whistler from Tayandu Islands) our result suggests that geographical isolation may be more important than plumage differences when making avian taxonomic decisions. Based on the genomic results, we propose that the oceanic/archipelagic *P. rufiventris xanthetraea* on Grande Terre, New Caledonia and *P. leucogastra meeki* should be recognised as full species, respectively, based on their genomic distinctness from their Sahul relatives and their isolated distributions. We further propose that *P. arctitorquis tianduana* be recognised as a presumably homoploid hybrid species, *P. tianduana*, based on its clearly admixed genomic content. Both *P. johni* and *P. l. leucogastra* are nested within *P. griseonota* and *P. monacha* , respectively. Therefore, *P. johni* Hartert, 1903 is best recognised as a subspecies within *P. griseonota* G.R. Grey, 1862, as Dickinson and Christidis (2014) and Clements et al. (2023) have done. Similarly, *P. l. leucogastra* Salvadori & D'Albertis, 1876 is best treated subspecifically within *P. monacha* G.R. Grey, 1858. The latter taxonomic change has not previously been proposed to our knowledge and would necessitate elevation of the only other subspecies currently in *P. leucogastra* , *P. leucogastra meeki* E. Hartert, 1898, to species rank as *P. meeki* . These rearrangements would lead to the recognition of eight species within this part of the broader *Pachycephala* complex. Conceivably, taxonomists drawing on additional lines of data may recognise further species‐level taxa within this complex. However, the well‐documented potential of young taxa to hybridise extensively when opportunities arise, and which is clear in this study, cautions the need for being restrictive when dividing evolutionarily young taxa into many taxonomic units, especially when they inhabit environmentally unstable regions.

## 5 Conclusions

This study adds to the growing knowledge that hybridisation is widespread in nature and that evolution in young species is often more akin to a reticulate network than to a bifurcating tree. The study also shows that introgressive hybridisation may be particularly pervasive in young species complexes occupying environmentally unstable regions. Plumage and vocal differences, often considered as strong pre‐zygotic barriers to gene flow in birds, may to a large extent not prevent gene flow during environmentally unstable conditions. In addition, this study highlights the challenges of estimating hybridisation patterns in systems with extensive histories of introgression when gene flow between taxa is not exclusively unidirectional.

## Author Contributions

M.I. and K.A.J. conceived the idea for the study. M.I., F.T. and I.A.M. carried out the laboratory work and I.A.M., F.T., B.G., J.A.A.N. and T.V. analysed the data with input from M.I. and K.A.J. M.I. drafted the manuscript and all authors provided feedback and agreed to the final version.

## Conflicts of Interest

The authors declare no conflicts of interest.

## Benefit Sharing Statement

The Nagoya Protocol is not applicable for the majority of the samples used in this study as they are old museum samples collected many decades ago. Fresh samples in this study are all from Australian National Wildlife Collection and have all been collected and shared in accordance with national laws and regulations.

## Supporting information


Figure S1.

## Data Availability

All raw sequencing data generated for this study have been deposited at the European Nucleotide Archive (accession number PRJEB81482) and all other metadata are present in Table 1. Mitochondrial genome sequences, FASTA‐format alignments used to construct the phylogenetic trees, and genotype calls for all samples across both autosome‐linked and Z chromosome‐linked contigs are deposited in DRYAD (https://doi.org/10.5061/dryad.qv9s4mwsk).
