# Supplementary material for: Reticulate and Hybrid Speciation is Promoted by Environmental Instability in an Indo‐Pacific Species Complex of Whistlers (Aves: Pachycephala)
Source: Mol Ecol. 2025 Jul 12;34(21):e70018. doi: 10.1111/mec.70018 (PMC12573736; doi:10.1111/mec.70018)
Supplement: Supplementary file 1 — Figure S1. [file MEC-34-e70018-s001.pdf]

Supplementary Material

Reticulate and hybrid speciation is promoted by environmental instability in an Indo-Pacific species complex of whistlers (Aves: *Pachycephala*)

Martin Irestedt[1], Ingo A Müller[1,2], Filip Thörn[1,2], Leo Joseph[3], Johan A A Nylander[1], Benjamin Guinet[1,5], Tom van der Valk[1,5,6], Knud Andreas Jønsson[1,4]

[1]*Department of Bioinformatics and Genetics, Swedish Museum of Natural History, SE-104 05, Stockholm, Sweden.*

[2]*Department of Zoology, Stockholm University, Stockholm, Sweden*

[3]*Australian National Wildlife Collection, National Research Collections Australia, CSIRO, Canberra, Australia*

[4]*Natural History Museum of Denmark, University of Copenhagen, DK-2100 Copenhagen Ø, Denmark*

[5]*Centre for Palaeogenetics, Svante Arrhenius väg 20C, 10691 Stockholm, Sweden.*

[6]*Science for Life laboratory, Stockholm*

Corresponding author: Martin Irestedt (martin.irestedt@nrm.se)

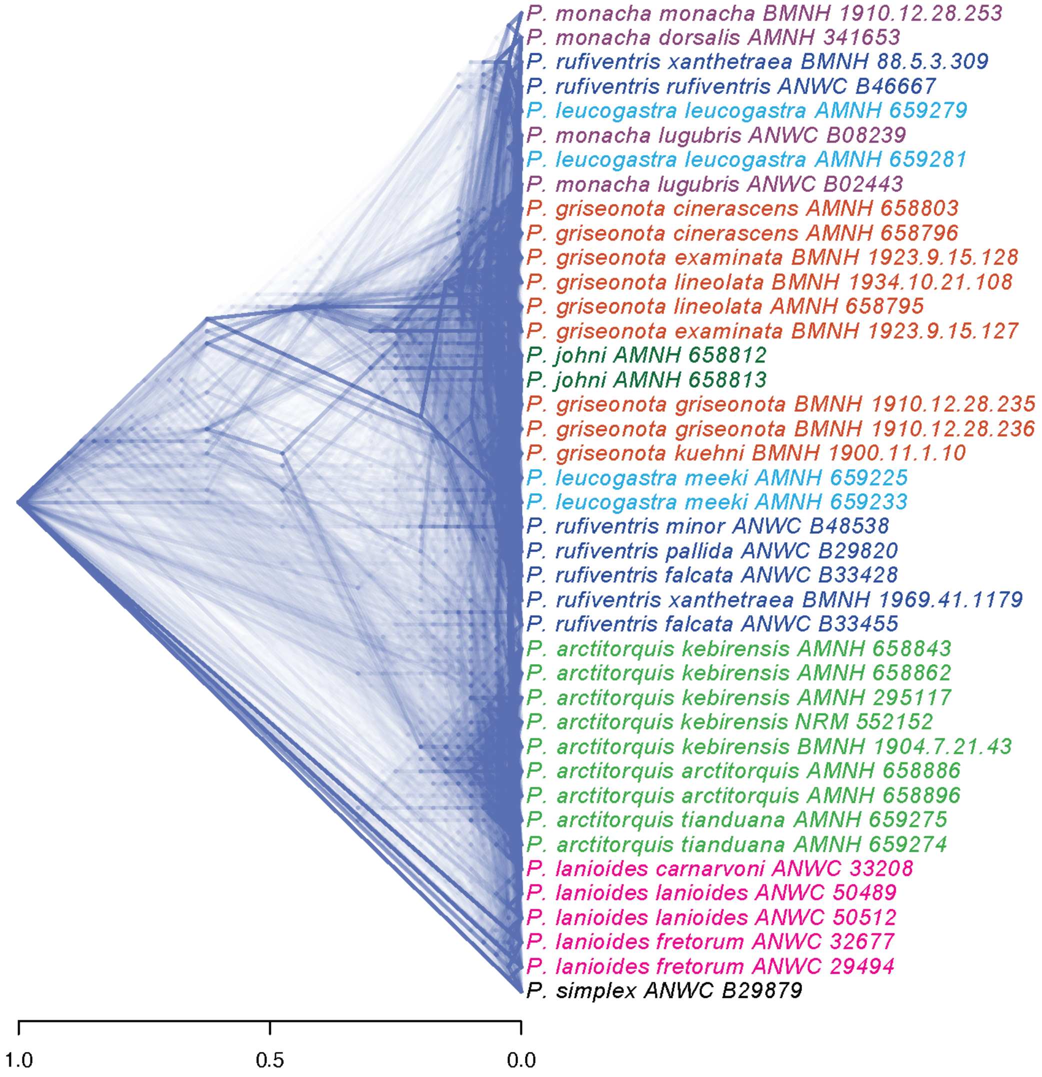

Figure S1. Autosomal cladogram that visualizes the reticulated evolutionary history of this whistler radiation. This cladogram, generated using the function densiTree from the R package phangorn, illustrates the topologies of 3,145 phylogenetic trees each composed of 10,000bp DNA alignment. The density and thickness of the blue lines visually convey the consensus and variability among the trees, highlighting the most supported branches in the dataset.

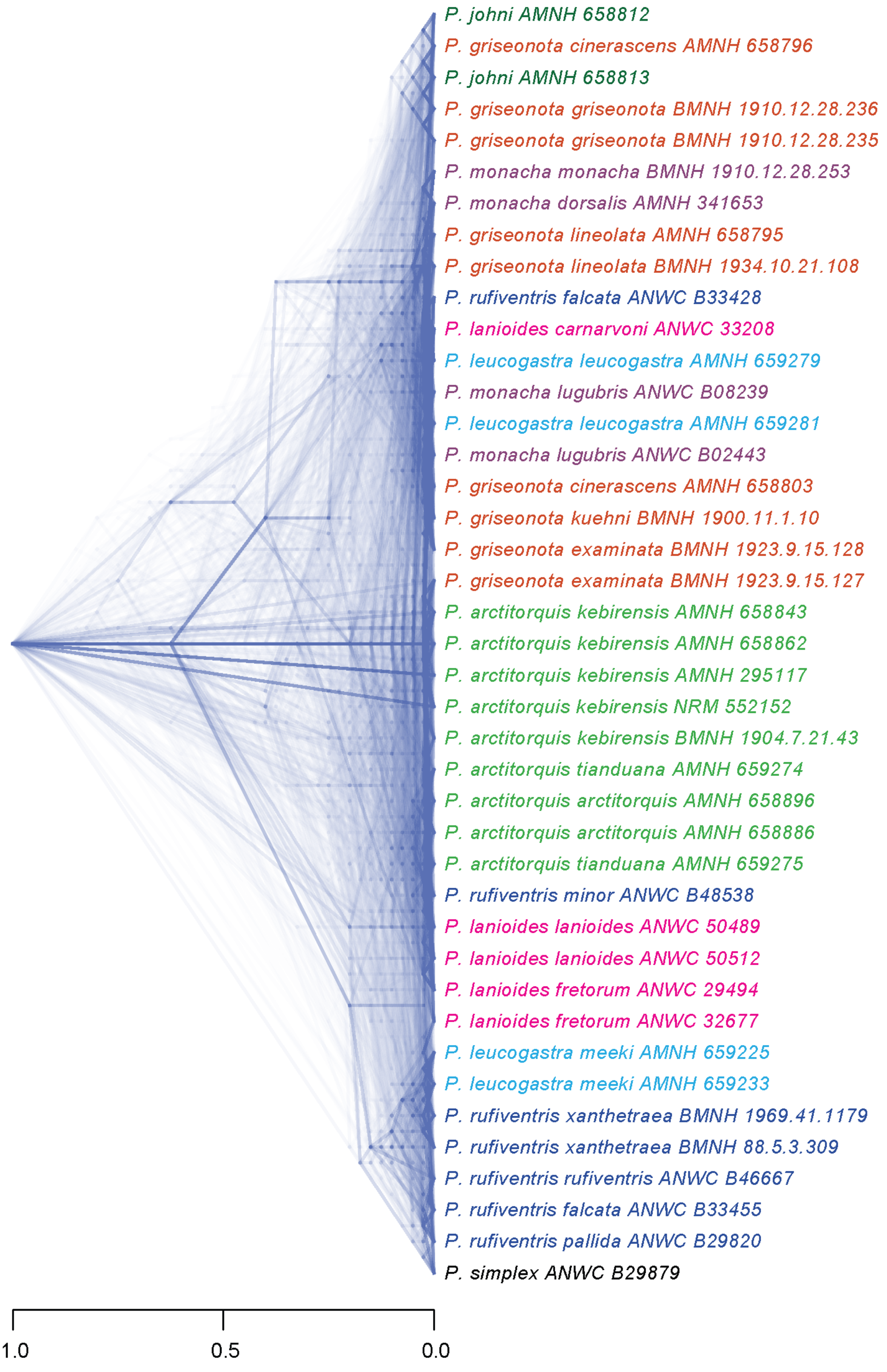

Figure S2. Z-chromosome cladogram that visualizes the reticulated evolutionary history of this whistler radiation. This cladogram, generated using the function densiTree from the R package phangorn, illustrates the topologies of 270 phylogenetic trees each composed of 10,000bp DNA alignment. The density and thickness of the blue lines visually convey the consensus and variability among the trees, highlighting the most supported branches in the dataset.

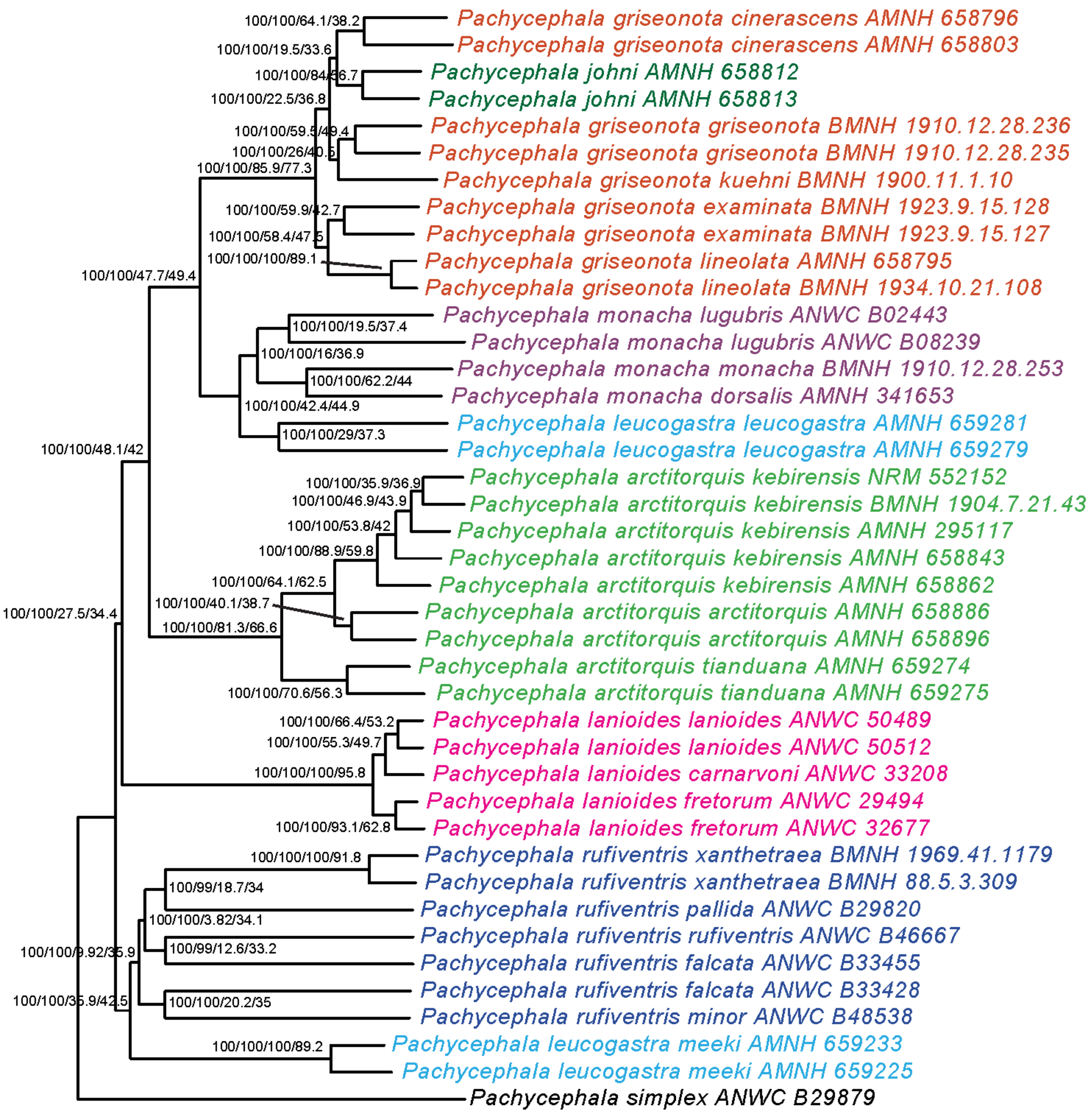

Figure S3. Z-chromosome phylogenetic tree. The tree was built using IQ-TREE (v 2) on 270 partitions of 10,000bp from the chromosome Z. The tree was rooted using *Pachycephala simplex* as outgroup. Support values at nodes are provided as follow UFBoot/aLRT/wCF/sCF.

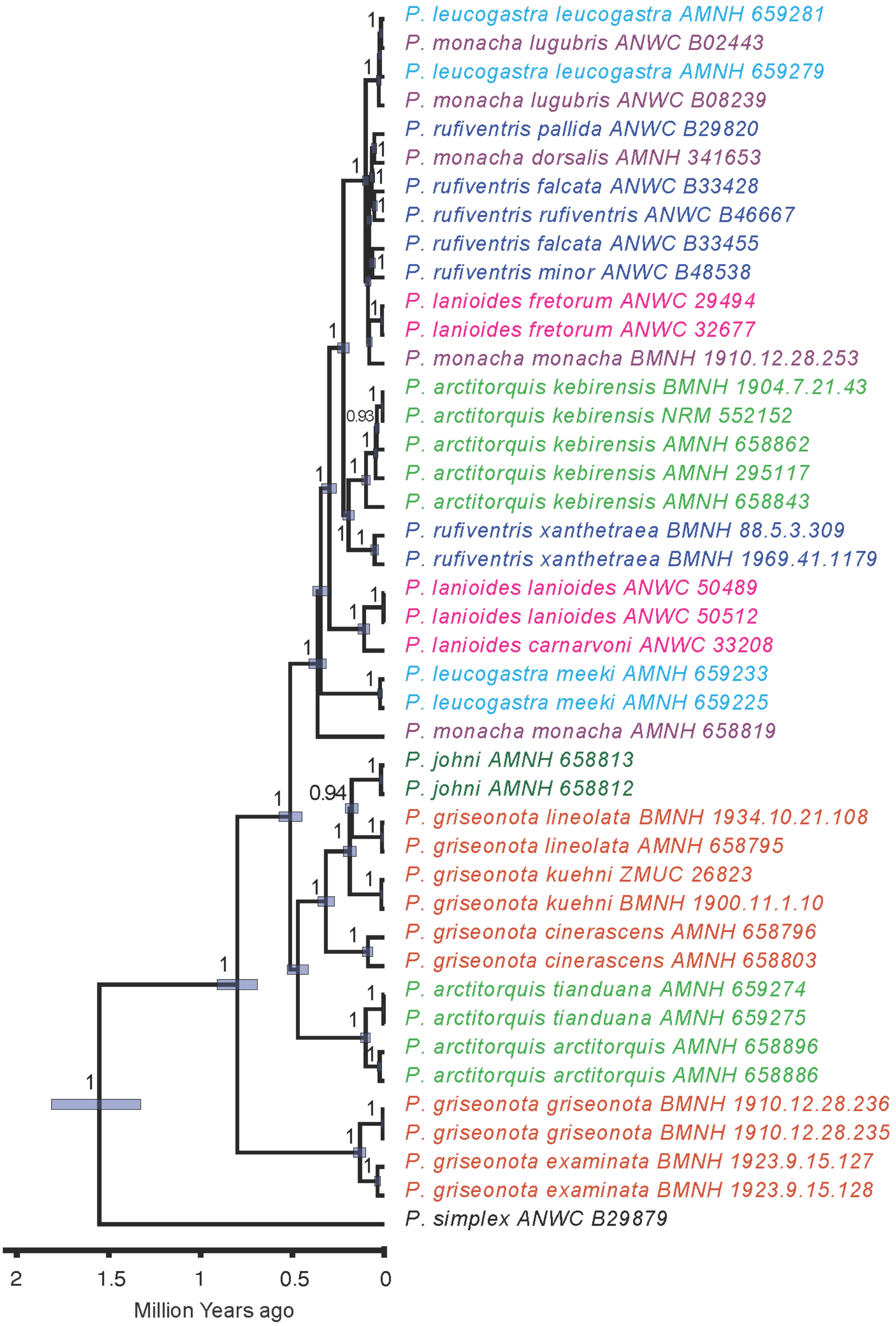

Figure S4. Dated mitochondrial tree. Scale at the bottom is in MYA.

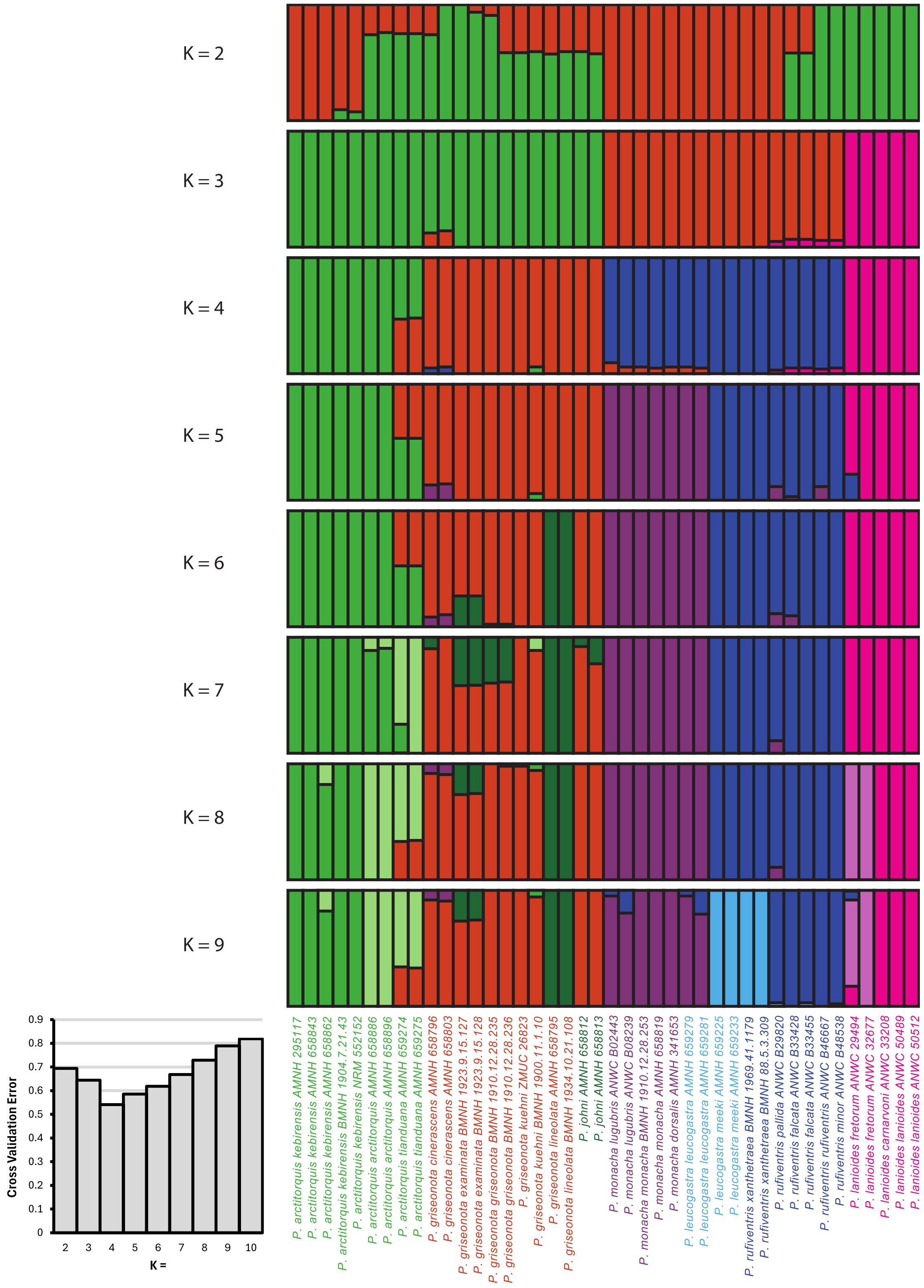

Figure S5. Admixure clustering based on genotype likelihoods (autosomes) for K 2-9. Cross validation errors for K 2-9 are shown at the bottom left. The admixture results are complex as signs of admixture vary depending on K-values. It is noteworthy, however, that Sahul taxa (including the two oceanic subspecies *P. rufiventris xanthetraea* and *P. leucogastra meeki*) cluster together at K=3 and K=4, but that they also contain components from other clusters that differ and that *P arctitorquis tianduana* is supported to be a hybrid population between the two species *P. arctitorquis* and *P. griseonota* at almost all K values.

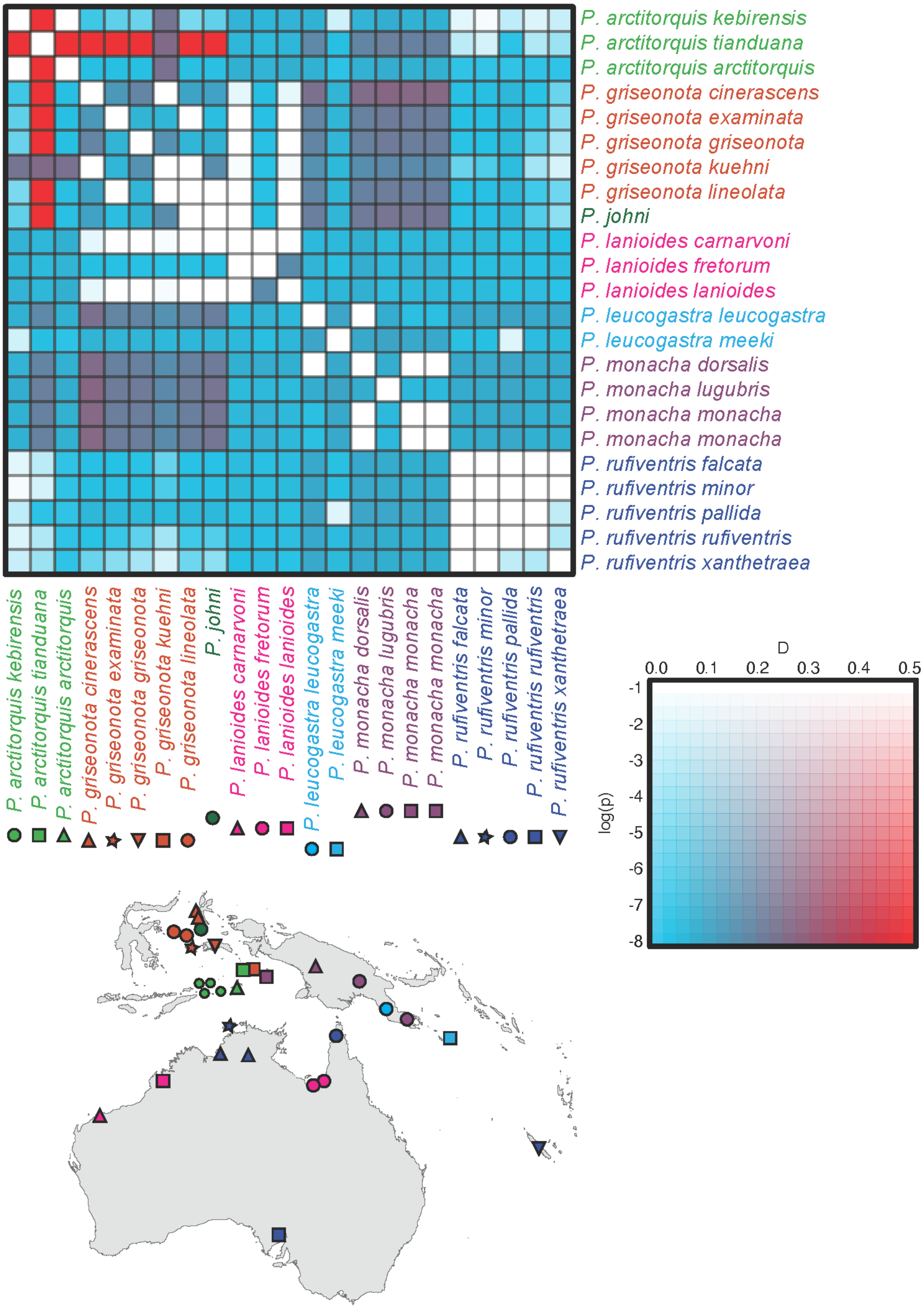

Figure S6. Heatmap showing patterns of gene flow between all (sub)species pairs using the ABBA-BABA test. The results show that *P. arctitorquis tianduana* has high signatures of allele sharing with P. *griseonota*/*P. johni*. We also recover a slight signature of allele sharing between P. rufiventris and *P. monacha*/*P. leucogastra leucogastra*, which is congruent with other analyses herein. The clear signatures of allele sharing between *P. griseonota*/*P. johni* and *P. leucogastra leucogastra*/*P. monacha* in this analysis is not evident in all other analyses in this study.

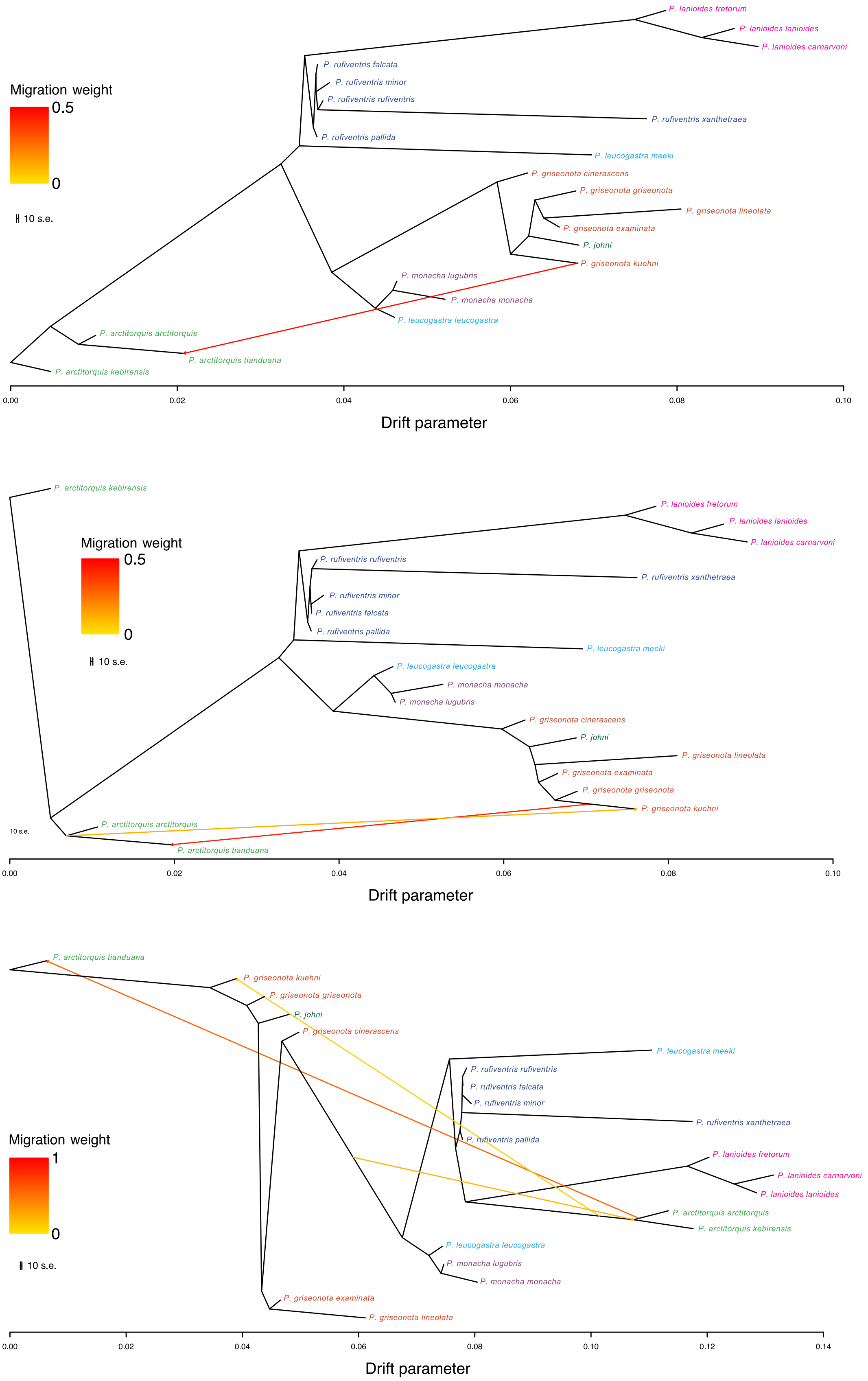

Figure S7. TreeMix analyses showing migration events 1-3 on unrooted maximum likelihood trees. The analysis suggests that gene flow between *P. griseonota kuehni* and *P. arctitorquis tianduana* is the most dominant and only gene flow between the *P. griseonota and P. arctitorquis* clades are supported.
